# Thermosonication-enhanced bioaccessibility and functional quality of dill juice: an *in vitro* digestion approach

**DOI:** 10.3389/fnut.2025.1666391

**Published:** 2025-09-22

**Authors:** Seydi Yıkmış, Abdullah Ateş, Selinay Demirel, Okan Levent, Nazlı Tokatlı, Nazan Tokatlı Demirok, Moneera O. Aljobair, Emad Karrar, Suleiman A. Althawab, Isam A. Mohamed Ahmed

**Affiliations:** ^1^Department of Food Technology, Tekirdag Namık Kemal University, Tekirdag, Türkiye; ^2^Department of Computer Engineering, Faculty of Engineering, Inonu University, Malatya, Türkiye; ^3^Nutrition and Dietetics, Faculty of Health Sciences, Tekirdag Namık Kemal University, Tekirdag, Türkiye; ^4^Department of Food Engineering, Faculty of Engineering, Inonu University, Malatya, Türkiye; ^5^Department of Computer Engineering, Faculty of Engineering and Natural Sciences, Istanbul Health and Technology University, Istanbul, Türkiye; ^6^Department of Sports Health, College of Sports Sciences and Physical Activity, Princess Nourah bint Abdulrahman University, Riyadh, Saudi Arabia; ^7^Department of Plant Sciences, North Dakota State University, Fargo, ND, United States; ^8^Department of Food Sciences and Nutrition, College of Food and Agricultural Sciences, King Saud University, Riyadh, Saudi Arabia

**Keywords:** thermosonication, *in vitro* digestion, bioaccessibility, dill juice, equilibrium optimizer (EO)

## Abstract

The effects of thermosonication — a non-thermal method considered an alternative to conventional thermal treatments — on the functional components in dill (*Anethum graveolens*) juice were comprehensively investigated in this study, as well as the post-digestion bioaccessibility levels of these components. In this study, samples from three different treatment groups— thermosonicated (TS-DJ), control (CDJ), and thermally pasteurized (P-DJ) were compared in terms of key parameters such as total chlorophyll content, total phenolic content (TPC), iron reducing capacity (FRAP), and *β*-carotene. Additionally, a controlled *in vitro* digestion system was used to analyze the stability and recovery rates of bioactive substances and volatile aroma compounds at different stages of the digestion process (gastric, oral, and intestinal phases). The findings revealed that thermosonication was highly effective in preserving the bioactive components both at the initial level and during the 21-day storage period and significantly increased their post-digestion bioaccessibility levels. Optimization of application parameters was achieved using a combination of Equilibrium Optimization algorithms and Response Surface Methodology (RSM), and the resulting prediction models were validated with high statistical confidence. The Pearson correlation analysis revealed significant positive correlations among *β*-carotene, characteristic volatile compounds, and total phenolic compounds. This suggests that the increase in these bioactive compounds may be directly related to the improvement in the aroma profile of dill juice. The data obtained indicate that thermosonication may offer an effective alternative to conventional thermal treatments in enhancing the functional quality of dill juice and its post-digestive bioaccessibility. However, further studies are needed to assess its potential for consumer acceptance and industrial integration. In this context, the study reveals important findings that will help develop new technologies for processing plant-based beverages.

## 1 Introduction

In recent years, interest in plant-derived products and medicines has been rapidly increasing. This is primarily because compounds derived from the flowers, leaves, stems, fruits, and roots of plants generally have lower toxicity and side effect profiles (1). In this context, dill (*Anethum graveolens*), an aromatic and fragrant plant belonging to the Apiaceae family, is widely used in both traditional and modern practices thanks to its nutraceutical and therapeutic properties (2). Dill is defined in two different forms: European dill (*Anethum graveolens*) and Indian dill (*Anethum sowa*) (3). This plant contains various biologically active compounds, including essential oils, fatty acids, polyphenols, carvone, and limonene. These compounds provide dill with antibacterial, anti-inflammatory, antioxidant, antihyperlipidemic, and antidiabetic effects (4–6). In addition, dill has a wide range of uses not only in traditional medicine and cuisine, but also in the formulation of antimicrobial and insect repellent agents, in the production of medicines and food supplements, in the beverage industry and in various pharmaceutical products (7).

In the field of food technology, innovative processing methods are being developed to meet consumers’ demands for healthy, delicious and longer shelf-life products (8). While traditional thermal processes, particularly pasteurization, ensure microbial safety, they can disrupt the structure of food components and lead to a decrease in sensory quality. This drawback has led to the rise of non-thermal methods, thermosonication, that can preserve nutritional value and sensory properties (9). The properties and quality of fruit and vegetable juices are particularly well-preserved by thermosonication, and it is considered a good alternative to traditional heat treatments (10). This technology enables higher preservation of the nutritional content of products by activating enzymes and providing microbial control at lower temperatures without the need for high-temperature applications such as pasteurization (11, 12).

On the other hand, a good understanding of the digestive system mechanisms is of great importance for the evaluation of functional products and the development of food processing techniques (13). Human digestive system experiments are limited due to ethical concerns and high costs. Therefore, the *in vitro* digestion model developed by the INFOGEST international consortium, simulating the mouth, stomach and small intestine stages, is considered an effective alternative method to evaluate the bioaccessibility of nutrients and digestibility of macronutrients by mimicking gastrointestinal processes (14, 15).

Surface response methodology (RSM), a statistical technique based on regression and variance analysis principles, is a method that provides optimization, improves and accelerates the product development process (16). Equilibrium Optimizer (EO), also known as meta-heuristic, is an easy-to-implement, powerful, and flexible algorithm for solving optimization problems (17). This study aimed to optimize the thermosonication and thermal pasteurization processes applied to fresh dill juice using RSM and EO methods for total chlorophyll and β-carotene content. Furthermore, we aimed to comprehensively examine the treatment effects by comparing functional properties such as total phenolic content, antioxidant capacity, volatile compound profile, and bioavailability assessed by an *in vitro* digestion model. A meticulously planned experimental design was implemented, and the reliability of the study results was ensured through replication and controls. The findings are expected to contribute scientifically to the development of sustainable and natural food processing technologies.

## 2 Materials and methods

### 2.1 Preparation of dill juice

This study used dill (*Anethum graveolens* L.) samples that were obtained from local producers operating in the Tekirdağ province of Türkiye. Samples were stored in a controlled environment at +4°C. In the preparation phase, the plant’s mature and stem tissues were removed. A Waring brand commercial blender (Model HGB2WTS3) was used for mechanical homogenization to ensure a uniform particle size distribution in the samples. The mixture was then filtered through Whatman No. 1 filter paper to remove cellulose-containing residues. Finally, the suspension was mixed with a vortex mixer at 2,000 rpm for 1 min to standardize the macromolecular distribution. Untreated dill juice was used as the control group (CDJ) in the experimental analyses.

### 2.2 Thermal pasteurization treatment

The samples, which had been prepared, were then transferred with care to 100-mL glass bottles. These bottles were then pasteurized at a temperature of 85° ± 1°C for a period of 2 min. This pasteurization process was carried out using a water bath (Wisd model WUC-D06H, Daihan, Wonju, Korea) that was temperature-controlled. This heat treatment was performed to reduce microbial load and increase product stability. Following pasteurization, the dill juice samples were gradually cooled to room temperature to prevent deterioration of their chemical components and then stored frozen at −20 ± 1°C until further analysis. Samples in this treatment group are designated “P-DJ” in this study.

### 2.3 Thermosonication treatment

Thermosonication was applied to dill juice samples (100 mL, P-DJ) using an ultrasonic probe-type system (UP200St, Hielscher Ultrasonics, Berlin, Germany) with and a nominal output power of 200 W and a frequency of 26 kHz. Process parameters were determined to be optimized as amplitude levels (60, 70, 80, 90, and 100%), application times (4, 6, 8, 10, and 12 min), and operating temperatures in fixed mode (40, 45, 50, 55, and 60°C). During ultrasonic energy application, an ice-water circulating cooling system was used to ensure that the samples remained within the target temperature range and to prevent thermal degradation. After the application, the samples subjected to thermosonication (TS-DJ) were placed in an ice bath for rapid cooling and then stored at −18 ± 1°C until further analyses.

### 2.4 Response surface methodology (RSM)

To evaluate the effects of thermosonication on *β*-carotene and total chlorophyll, dill juice samples were analyzed by Response Surface Methodology (RSM) using Minitab Statistical Analysis Software (version 18.1.1). Within the scope of optimization studies, applications were carried out under 15 different experimental conditions, and these conditions are presented in Table 1. The suitability of the model was evaluated according to the analysis of variance (ANOVA) results, adjusted *R*^2^ and *R*^2^ coefficients, and significance tests (Table 2). The independent variables were defined as follows: time (in minutes, *X*_1_), amplitude (% of *X*_2_), and temperature (in °C, *X*_3_). The dependent variables were determined as the levels of β-carotene and total chlorophyll. The second-degree polynomial equation given below was used in creating the model (Equation 1).


(1) $$y=\beta_{0}+\sum_{i=1}^{3}\beta_{i}X_{i}+\sum_{i=1}^{3}\beta_{\mathrm{ii}}X_{i}^{2}+\sum_{\begin{array}{c} i=1\\ i<j \end{array}}^{3}\sum_{j=1}^{3}\beta_{\mathrm{ij}}X_{i}X_{j}$$

**Table 1:** Effects of ultrasonic time, temperature, amplitude, and on β-carotene and total chlorophyll content in dill juice treated with thermosonication.

Run no.	Independent variables			Dependent variables					
	Time (*X*_1_) (min)	Amplitude (*X*_2_) (%)	Temperature (*X*_3_) (°C)	β-carotene (mg/100 mL)			Total chlorophyll (g/100 mL)		
				Experimental data	RSM predicted	EO predicted	Experimental data	RSM predicted	EO predicted
1	16	60	50	29.56	29.46	29.41	2.9	2.92	2.90
2	12	60	40	33.83	33.55	33.60	3.33	3.31	3.34
3	12	60	60	34.76	34.69	34.64	3.3	3.30	3.29
4	16	80	60	31.32	31.54	31.61	2.96	2.94	2.95
5	8	100	50	31.08	31.20	31.18	2.84	2.82	2.80
6	8	80	60	35.14	34.73	34.66	3.14	3.13	3.13
7	16	100	50	31.93	31.43	31.42	2.91	2.91	2.90
8	12	80	50	38.14	38.37	38.33	3.36	3.39	3.37
9	12	80	50	38.73	38.37	38.33	3.41	3.39	3.37
10	8	60	50	34.31	34.83	34.81	3.37	3.37	3.37
11	12	100	60	32.37	32.67	32.72	2.88	2.90	2.93
12	12	100	40	33.84	33.93	33.89	3.15	3.15	3.13
13	8	80	40	34.37	34.17	34.26	3.22	3.24	3.26
14	16	80	40	31.79	32.22	32.14	3.07	3.07	3.07
15	12	80	50	38.22	38.37	38.33	3.41	3.39	3.37
(RSM optimization parameters)	11.15	77.69	50.30	38.51	38.53		3.41	3.42	
Experimental values					41.14 ± 1.39		3.52 ± 0.07		
% Difference					6.34%		3.12%		
(Equilibrium optimization parameters)	11.00	74.59	49			38.39			3.42
Experimental values						41.45 ± 1.87			3.52 ± 0.03
% Difference						7.38%			2.92%

*X*_1_, time (min); *X*_2_, amplitude (%); *X*_3_, temperature (s); RSM, response surface methodology; EO, equilibrium optimization.

**Table 2:** ANOVA results for RSM modeling of β-carotene and total chlorophyll content in thermosonicated dill juice.

Source	DF	β-carotene (mg/100 mL)		Total chlorophyll (g/100 mL)	
		*F*-value	*P*-value	*F*-value	*P*-value
Model	9	42.94	0.000	74.36	0.000
Linear	3	17.83	0.004	91.99	0.000
*X*_1_	1	48.64	0.001	72.54	0.000
*X*_2_	1	4.81	0.080	170.74	0.000
*X*_3_	1	0.03	0.877	32.68	0.002
Square	3	99.16	0.000	99.33	0.000
*X*_1_ *X*_1_	1	174.96	0.000	208.86	0.000
*X*_2_ *X*_2_	1	125.92	0.000	103.47	0.000
*X*_3_ *X*_3_	1	35.29	0.002	18.55	0.008
2-Way Interaction	3	11.82	0.010	31.77	0.001
*X*_1_ *X*_2_	1	28.76	0.003	79.38	0.000
*X*_1_ *X*_3_	1	1.41	0.288	0.25	0.642
*X*_2_ *X*_3_	1	5.28	0.070	15.68	0.011
Error	5				
Lack-of-fit	3	3.77	0.217	1.17	0.492
Pure error	2				
Total	14				
*R* ^2^		98.72%		99.26%	
Adj. *R*^2^		96.42%		97.92%	
Pred. *R*^2^		82.2%		91.84%	

*R*^2^, coefficient of determination; *p* < 0.01, very significant differences; *p* < 0.05, significant differences; DF, desgress of freedom; *X*_1_, Time; *X*_2_, amplitude; *X*_3_, temperature.

In the model equation, *Y* represents the dependent variable to be estimated in the study. *β*₀ is the constant term of the model and represents the theoretical value of *Y* when all independent variables are zero. The *β*ᵢ coefficients represent the linear (first-order) effect of each independent variable (*X*ᵢ) on *Y*, while the *β*ᵢᵢ coefficients represent the curvilinear or second-order effects of the same variables on *Y*. Conversely, the *β*ᵢⱼ coefficients explain the joint effect of the interaction between two different independent variables (*X*ᵢ and *X*ⱼ) on the dependent variable. Thanks to this structure, the contributions of both individual and interacting variables to the system can be evaluated holistically.

### 2.5 Equilibrium optimizer (EO)

The equilibrium optimization algorithm draws inspiration from dynamic mass balance within the control volume. According to general mass balance (Equation 2), the change in mass with time is obtained by subtracting the total mass entering the system from the total mass leaving the system (18).


(2) $$V\frac{\mathrm{dC}}{\mathrm{dt}}=QC_{\mathrm{eq}}-\mathrm{QC}+G$$

where *C* shows concentration (*V*), $V\frac{\mathrm{dC}}{\mathrm{dt}}$ the rate of change of mass within the control volume, *Q* flow rate, *C*_eq_ equilibrium concentration, G rate of mass change. Equation 3 shows the particle update rule. Each particle operates independently of the others according to these three conditions to update its concentration (18).


(3) $$F=\exp[-\lambda (t-t_{0})]$$

where *t*_0_ and *C*_0_ show the initial time and concentration, respectively. The first stage (concentration equilibrium) represents one of the best solutions selected randomly from the pool. The second stage signifies the disparity in concentration—or density—between the current particle and the equilibrium state. The generation rate is the focus of the third stage (18).

The initial concentration for Equilibrium Optimization is generated using a uniform random distribution based on the number of particles. The initial population is generated as follows (Equation 4):


(4) $$C_{i}^{\text{initial}}=C_{\min}+{\mathrm{rand}}_{i}(C_{\min}-C_{\max})\;i=1,2,3,\ldots .n$$

where $C_{i}^{\text{initial}}$ shows the concentration vector for each particle $C_{\min}\;$ and $\text{Csho}w_{\max}$ shows maximum and minimum values. rand_i_, n shows a random number between [0, 1] and the number of the particle in the population, respectively.

Particle evaluation is performed according to the defined objective function, and these values are used as the basis for determining potential equilibrium solutions. The resulting equilibrium position represents the algorithm’s final solution state, and this point is defined as the global optimum. This optimization algorithm is capable of generating four different, near-optimal solution alternatives under various problem types and conditions. During the optimization process, the average of the candidate solutions is considered, and each particle is selected randomly from among these candidates. The Equilibrium Optimization Algorithm ensures efficient scanning of the solution space by establishing an effective balance between the exploration and exploitation phases throughout the algorithm’s execution. The algorithm’s update mechanism is defined within the framework of the rules specified below Equation 5 (18).


(5) $$\vec{C}={\vec{C}}_{\mathrm{eq}}+(\vec{C}-{\vec{C}}_{\mathrm{eq}})\vec{F}+\frac{\vec{G}}{\vec{\lambda }V}(1-\vec{F})$$

**Figure 1:**
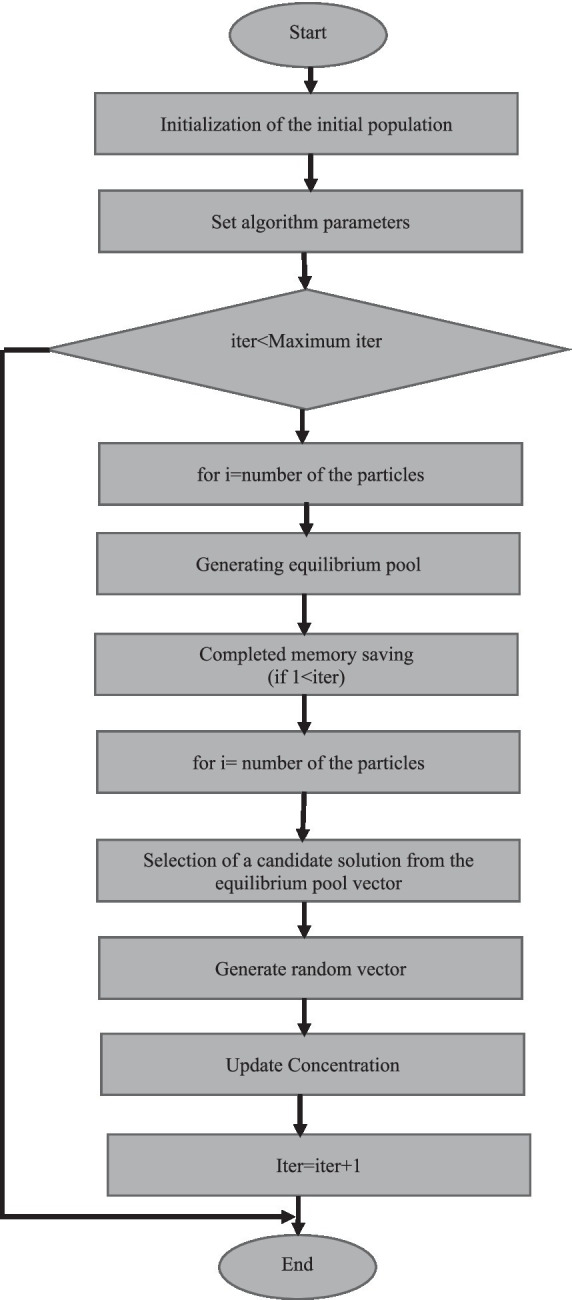
Flow chart of the equilibrium optimization method.

The flowchart of the Equilibrium Optimization algorithm is given as follows. In this study, the proposed quadratic model parameters are optimized according to the given flowchart (Figure 1).

### 2.6 Determination of bioactive compounds

The total phenolic content of the samples was determined quantitatively by colorimetric analysis with Folin–Ciocalteu reagent, and the results were expressed as gallic acid equivalent in milligrams per liter (mg GAE/L) (19). A quantitative analysis of chlorophyll content was performed on the samples using a widely used spectrophotometric method for plant materials developed by Hiscox and Israelstam (20). A solution consisting of 3 mL of dill extract and 3 mL of 80% (v/v) acetone was homogeneously mixed. The resulting mixture was filtered three times through Whatman filter paper to increase the optical purity of the sample. The light absorption values of the filtrate were then measured spectrophotometrically at 645 nm and 663 nm for chlorophyll determination. The FRAP (Ferric Reducing Antioxidant Power) method was used to determine the total antioxidant capacity of the dill juice samples. The optical density of the colored complex formed by the reduction of ferric ions (Fe^3+^) to the ferrous form (Fe^2+^) is the basis of this method. The complex’s absorptivity was measured at 593 nm, and its antioxidant capacity was calculated based on these findings. The calibration curve was generated using Trolox, a synthetic antioxidant analog. The findings are presented in millimoles of Trolox equivalents per liter of dill juice (mmol TE/L) (21).

A minor modification of a spectroscopic method was used to determine the total carotenoid content of dill juice samples (22, 23). In analyses to determine total carotenoid content, 1 mL of dill juice was mixed with 5 mL of a methanol solution prepared at a 1:2 ratio. The prepared mixture was allowed to stand until the phases were completely separated, and then the upper phase was carefully removed. 0.5 mL of saturated sodium chloride solution was added to the upper phase to restore homogeneity. The lower phase was subsequently treated with anhydrous sodium sulfate. The solution was centrifuged at 4,000 rpm for 10 min to increase its clarity (GYROZEN 1730 R, Korea). The upper phase obtained after centrifugation was collected again and diluted with 5 mL of methanol solution before analysis. Absorption measurements of the prepared extract were carried out using a UV–Visible spectrophotometer operating at 450 nm wavelength (SP-UV/VIS-300SRB, Spectrum Instruments, Victoria, Australia). The measured absorbance values were compared to the calibration curve created using β-carotene standard solutions, and the results were calculated as the amount of β-carotene in milligrams per liter of dill juice.

#### 2.6.1 Determination of phenolic compounds

Determination of phenolic compounds was carried out using an Agilent 1,260 Infinity high-performance liquid chromatography system equipped with a diode array detector (DAD). Analyses were performed using a C18 Agilent column with 250 × 4.6 mm dimensions and 5 μm particle diameter, according to the protocol described by Portu et al. (24). The column temperature was kept constant at 30°C, and the flow rate of the mobile phase was set at 0.80 mL/min. Determination of phenolic compounds was carried out at wavelengths of 280 nm, 320 nm, and 360 nm. The concentration values obtained were reported as mg gallic acid equivalent (mg GAE) per 100 mL sample volume. Analyses are presented as the mean values obtained from triplicate measurements each.

### 2.7 Determination of volatile compounds

Before analysis, 5 μL of cyclohexanone was mixed with the dill juice samples as an internal standard, followed by headspace solid-phase microextraction (HS-SPME). The extraction process was initiated by transferring 10 mL of the sample to a 22 mL crimp-cap headspace vial. A 50/30 μm DVB/CAR/PDMS (Supelco, Bellefonte, PA, United States) fiber type was used in all analyses. Samples were pre-incubated at 50°C for 10 min to increase the release of volatile compounds, followed by the extraction of volatile compounds from the headspace of the vial at the same temperature for 20 min. Desorption on the fiber was performed at 250°C for 1 min in split-injection mode. The separation of volatile compounds was equipped with an Agilent 7,890 Gas Chromatography system (Agilent Technologies, Santa Clara, CA, United States) and a 5,977 N Mass Spectrometer detector. The analysis was performed using a DB-5 column (Agilent Technologies). This column has a length of 30 m, a diameter of 0.25 mm at its inner core, and a film thickness of 0.25 μm. The column oven temperature was increased from an initial temperature of 40–230°C at a rate of 7°C/min, and then to 260°C at a rate of 100°C/min. It was maintained at this temperature for 11.7 min, resulting in a total analysis time of 60 min. Helium was used as the carrier gas with a flow rate of 1.2 mL/min. The injection port, ionization source, and transfer line were fixed at 250, 230, and 280°C, respectively. Mass spectra were recorded in the 40–350 m/z range with an ionization energy of 70 eV. Data were collected using ChemStation G1701 AA software (Hewlett-Packard, Palo Alto, CA, United States). Blank checks were performed between each sample run to prevent fiber contamination during analysis, and three replicate analyses were performed on a DB-5 column. Volatile compounds were identified by comparing the resulting mass spectra with the NIST/EPA/NIH Mass Spectral Library (version 2.0d; National Institute of Standards and Technology, Gaithersburg, MD, United States). Semi-quantitative determination of volatile compounds in each sample was performed using the internal standard method.

### 2.8 *In vitro*-simulated gastrointestinal digestion analysis

An *in vitro* digestion model was employed, followed by dialysis, according to the method described by Minekus et al. (14). The study used a three-phase methodology: an oral phase (pH 7.0 with *α*-amylase), a gastric phase (pH 3.0 with pepsin), and an intestinal phase (pH 7.0 with fresh bile and pancreatin). Following completion of the gastric and intestinal phases, β-carotene (mg/100 mL), antioxidant capacity (FRAP), total chlorophyll (g/100 mL), and total phenolic compounds (TPC) analyses were performed on the digested samples. For each treatment, measurements were made in three independent replicates, and the averages were evaluated.

### 2.9 Statistical analysis

To ensure the accuracy and reproducibility of the experimental procedures, each experiment was performed with three technical replicates. Statistical analyses were performed using one-way analysis of variance (ANOVA) to compare within-group variances, and Tukey’s HSD (Honestly Significant Difference) *post hoc* test was applied to determine between-group differences at a significance level of *p* < 0.05. Analyses were conducted using the SPSS statistical package program (version 22.0; SPSS Inc., Chicago, IL, United States). Data are presented as mean ± standard deviation (SD).

## 3 Results and discussion

### 3.1 Evaluation of results obtained with RSM and equilibrium optimizer (EO)

This study was optimized to analyze the effects of thermosonication treatment on the functional components of dill juice. The effects of three basic process parameters, namely duration (*X*_1_), amplitude (*X*_2_), and temperature (*X*_3_), on the β-carotene and total chlorophyll content were modeled and optimized using Response Surface Methodology (RSM). The effect of dill juice on the β-carotene (Equation 6) and total chlorophyll (Equation 7) content is presented in the following equations.


(6) $$\begin{array}{l} ß-\text{carotene}\;\mathrm{RSM}\;(\frac{\mathrm{mg}}{100\mathrm{mL}})=-77.3+4.057\;X_{1}+1.139\;X_{2}\\ +1.944\;X_{3}-0.2246\;X_{1}X_{1}-0.007623\;X_{2}X_{2}-0.01614\;X_{3}X_{3}\\ +0.01750\;X_{1}X_{2}-0.00775\;X_{1}X_{3}-0.00300\;X_{2}X_{3} \end{array}$$

(7) $$\begin{array}{l} \text{Total Chlorophyll}\;\mathrm{RSM}(\frac{g}{100\mathrm{mL}})=-1.475+0.1934\;X_{1}\\ +0.05192\;X_{2}+0.0880\;X_{3}-0.014245\;X_{1}X_{1}\\ -0.000401\;X_{2}X_{2}-0.000679\;X_{3}X_{3}+0.001688\;X_{1}X_{2}\\ -0.000187\;X_{1}X_{3}-0.000300\;X_{2}X_{3} \end{array}$$

Comparison of the experimental and estimated values in Table 1 reveals that the RSM model achieves high accuracy in terms of both dependent variables. In particular, in the parameter combination determined as optimum conditions as *X*_1_ = 11.15 min, *X*_2_ = 77.69%, *X*_3_ = 50.30 °C, β-carotene content was predicted as 38.53 mg/100 mL and total chlorophyll content as 3.42 g/100 mL. A study on African mango juice shows that thermosonication treatment results in a significant increase in carotenoid content, similar to our study (25). In another study, Maoto and Jideani (26) emphasized that the application of thermosonication process to watermelon juice preserves the ß-carotene content under optimal conditions. Another study reports that the application of optimum thermosonication conditions has the potential to increase and preserve carotenoid content in hog plum juice (27). The fact that these values show high agreement with experimental validation studies reinforces the reliability of the model.

Experimental repetitions performed under the conditions obtained as a result of RSM optimization yielded results that were even superior to the model’s predictions: β-carotene content was measured at 41.14 ± 1.39 mg/100 mL, and total chlorophyll content was measured at 3.52 ± 0.07 mg/100 mL. The difference between the predicted values and the experimental results is quite low, 6.34% for β-carotene and 3.12% for chlorophyll. These differences are within the acceptable range in food matrices, indicating that the model successfully represents the real process. Moreover, the low standard deviations obtained confirm the repeatability of the process and also reveal the applicability of the proposed optimization. These results emphasize that the RSM approach is a valid tool not only mathematically but also in terms of practical applications.

The ANOVA findings presented in Table 2 show that the models developed for both dependent variables have a high level of statistical significance (*p* < 0.001). The model *F*-values for β-carotene and total chlorophyll were calculated as 42.94 and 74.36, respectively. Especially the square terms (*X*_1_^2^, *X*_2_^2^, *X*_3_^2^) were found to be significant with very high F-values (e.g., *β*-carotene *F* = 174.96, *p* < 0.001 for *X*_1_^2^), which shows that the model is sensitive to the curvilinear structure and can reach the maximum-minimum points effectively. In terms of main effects, duration (*X*_1_) stands out as the most effective variable for both β-carotene and chlorophyll. While amplitude (*X*_2_) has a powerful impact on chlorophyll (*F* = 170.74, *p* < 0.001), it has a more limited effect on β-carotene. On the other hand, temperature (*X*_3_) had a significant effect only on chlorophyll, while it was found to be statistically insignificant for β-carotene (*p* = 0.877).

In terms of binary interactions, the combination of *X*_1_*X*_2_ (duration and amplitude) shows a significant effect on both bioactive components (*F* = 28.76 for β-carotene, *F* = 79.38 for chlorophyll; *p* < 0.01). This synergistic interaction shows that these two parameters should be considered together in determining the optimum process conditions. On the other hand, *X*_1_*X*_3_ and *X*_2_*X_3_ interactions have more limited and variable effects. Statistical parameters for the fit of the model are also exceptionally high: *R*^2^ = 98.72% for β-carotene, *R*^2^ = 99.26% for chlorophyll; adjusted *R*^2^ values are 96.42 and 97.92%, respectively. The fact that the predicted *R*^2^ values are above 80% indicates that the model not only fits the data but also has high predictive ability for new data. The findings indicate that thermosonication is a viable method for optimizing and maximizing bioactive components. Furthermore, the model can serve as a reliable guide in designing functional beverages. Consistent with the findings of our study, a robust correlation was identified between the experimental data and the data predicted by RSM in a study conducted on kinnow fruit juice, a product of RSM optimization. The study also shows that there is potential to maintain juice yield, phenolic content, and nutritional quality under optimum conditions (28).

In addition, the system converges to a quadratic model with 10 variables. Therefore, these parameters can be optimized using a different optimization algorithm, such as RMS, to demonstrate the reliability and repeatability of the results.

As is well known, heuristic and metaheuristic optimization algorithms are employed in solving various engineering problems. These methods can produce more stable results than other optimization algorithms in cases where there is model uncertainty.

Metaheuristic optimization algorithms are widely used in food engineering due to their ability to solve complex, non-linear, and multi-objective problems. These algorithms can produce practical solutions to issues that traditional optimization methods cannot solve by imitating natural processes. Therefore, meta-heuristic optimization algorithms are used in many areas in Food Engineering. Metaheuristic algorithms are widely used in optimizing food processing processes. For example, optimization of process parameters for vacuum belt drying of Citri Reticulatae Pericarpium using Particle Swarm Optimization (PSO) and Genetic Algorithm (GA) was presented in (29).

Another important area of use is formulation design. The optimization of production planning problems from multiple bakery product lines has been achieved through the implementation of Pareto-based multi-objective optimization algorithms, the non-dominated sorting genetic algorithm (NSGA-II), and the random search algorithm (30).

An integrated and flexible model has been developed for sustainable retail food supply network design, which takes into account route disruptions and traffic conditions, and reduces costs and CO2 emissions with dynamic pricing and multi-objective optimization methods (Utility Function Genetics Algorithm and Heuristic Multi-Choice Goal Programming) (31).

In this study, the Equilibrium Optimization (18). An algorithm was used. The Equilibrium Optimization (EO) algorithm is a meta-heuristic optimization technique inspired by the concepts of mass balance and dynamic balance. Models the process of reaching equilibrium in the control volume system, simulating the dynamic interactions of resources and expenses, and directs the search to reach the optimal solution (18).

Using this method, simultaneous optimization was performed for total Chlorophyll and ß-carotene, and the results were presented comparatively (Equations 8 and 9).


(8) $$\begin{array}{l} \text{Total Chlorophyll}(\frac{g}{100\mathrm{mL}})=-1.475+0.1934\;X_{1}\\ +0.05192\;X_{2}+0.0880\;X_{3}-0.014245\;X_{1}X_{1}\\ -0.000401\;X_{2}X_{2}-0.000679\;X_{3}X_{3}+0.001688\;X_{1}X_{2}\\ -0.000187\;X_{1}X_{3}-0.000300\;X_{2}X_{3} \end{array}$$

(9) $$\begin{array}{l} ß-\text{carotene}\;(\frac{\mathrm{mg}}{100\mathrm{mL}})=a11+a12\;X_{1}+a13\;X_{2}+a14\;X_{3}\\ +a15\;X_{1}X_{1}+a16\;X_{2}X_{2}+a17X_{3}X_{3}+a18\;X_{1}X_{2}\\ +a19\;X_{1}X_{3}+a20\;X_{2}X_{3} \end{array}$$

The objective function used during the optimization process is as follows. First, the following model is established for the Total Chlorophyll data. According to the input values given in the Total Chlorophyll predictive table, the values of a1, a2, …, a10 are derived with the Equilibrium optimization algorithm and placed in the relevant (Equation 10).


(10) $$\begin{array}{l} \text{Total Chlorophyll predic}(\frac{g}{100\mathrm{mL}})=a(1)*\text{input}(1,:)\\ +a(2)\text{input}(2,:)+a(3)\text{input}(3,:)+a(4)\text{input}{\left(1,:\right)}^{2}\\ +a(5)\text{input}{\left(2,:\right)}^{2}+a(6)\text{input}{\left(3,:\right)}^{2}+a(7)\text{input}(1,:)\\ \text{input}(2,:)+a(8)\text{input}(1,:)\;\text{input}(3,:)+a(9)\text{input}(2,:)\;\\ \text{input}(3,:)+a(10) \end{array}$$

Similarly, the ß-carotene predictive equation is obtained as follows (Equation 11).


(11) $$\begin{array}{l} ß-\text{carotene predic}\;(\frac{\mathrm{mg}}{100\mathrm{mL}})=a(11)*\text{input}(1,:)\\ +a(12)\text{input}(2,:)+a(13)\text{input}(3,:)+a(14)\text{input}{\left(1,:\right)}^{2}\\ +a(15)\text{input}{\left(2,:\right)}^{2}+a(16)\text{input}{\left(3,:\right)}^{2}+a(17)\text{input}(1,:)\\ \text{input}(2,:)+a(18)\text{input}(1,:)\;\text{input}(3,:)+a(19)\text{input}(2,:)\\ \;\text{input}(3,:)+a(20) \end{array}$$

Using these values, the objective function is created according to the MSE function as follows (Equations 12-14).


(12) $$\mathrm{MSE1}=\text{mean}({\left(\text{Real}\;\mathrm{Out1}–\text{Total Chlorophyll predic}\right)}^{2})$$

(13) $$\mathrm{MSE2}=\text{mean}({\left(\text{Real}\;\mathrm{Out2}–ß-\text{carotene predic}\right)}^{2})$$

(14) Objective function=mean(MSE1+MSE2)

The models obtained as a result of optimization are as follows (Equations 15 and 16):


(15) $$\begin{array}{l} \text{Total Chlorophyll}\;\mathrm{opt}(\frac{g}{100\mathrm{mL}})=-0.1295+0.1698X_{1}\\ +0.0428X_{2}+0.054X_{3}-0.014X_{1}X_{1}-0.0003826X_{2}X_{2}\\ -0.0004407X_{3}X_{3}+0.001784X_{1}X_{2}\\ -0.00000250027X_{1}X_{3}-0.000201795X_{2}X_{3} \end{array}$$

(16) $$\begin{array}{l} ß-\text{carotene}\;\mathrm{opt}\;(\frac{\mathrm{mg}}{100\mathrm{mL}})=-74.700+3.9592+1.1291X_{2}\\ +1.8774X_{3}-0.22404X_{1}X_{1}-0.007603X_{2}X_{2}+\\ -0.015722X_{3}X_{3}+0.01766X_{1}X_{2}\\ -0.006355X_{1}X_{3}-0.0029X_{3} \end{array}$$

**Figure 2:**
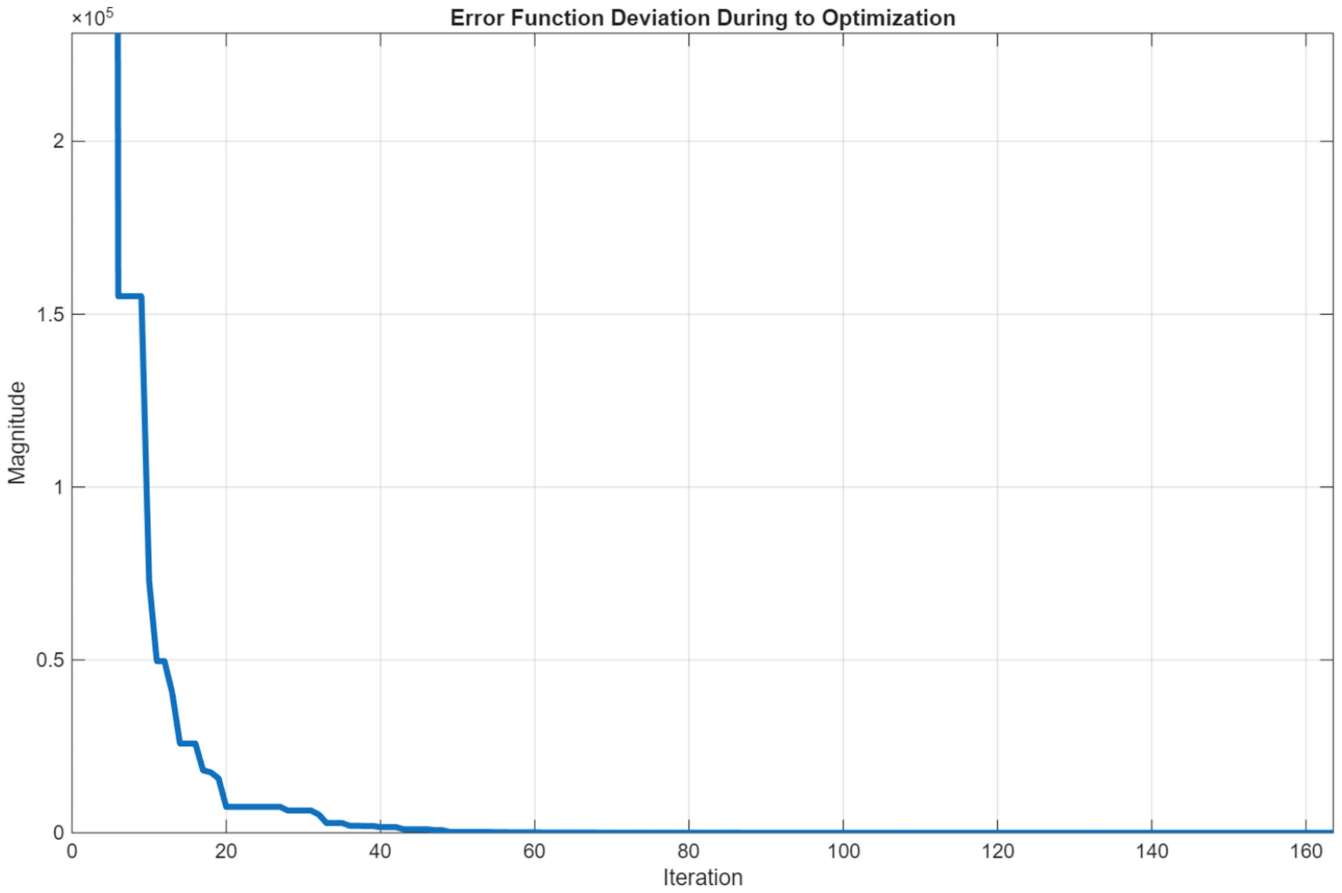
Error function deviation during to equilibrium optimization.

The error function change during optimization is as follows:

As shown in Figure 2, a very high decrease in the error function is observed in the first iterations. This shows that the algorithm can find better solutions quickly at the beginning and a good initial parameter selection is made. The decrease slows down in the following iterations. This shows that the algorithm now progresses with smaller improvements and approaches the optimum solution. After approximately 30–40 iterations, the error function remains almost constant from iteration to iteration. At this point, the algorithm is very close to the local or global minimum and cannot provide any further improvement. After approximately 60 iterations, the change has reached a negligible level. As can be seen, the Equilibrium optimization algorithm converges quite quickly. The fact that the error function remains constant after the 60th iteration shows that the solution is stable and the algorithm works well.

Then, the obtained optimization algorithm outputs are presented in comparison with the calculated RSM algorithm outputs and the actual outputs obtained from the system.

### 3.2 RSM and EO comparison

**Figure 3:**
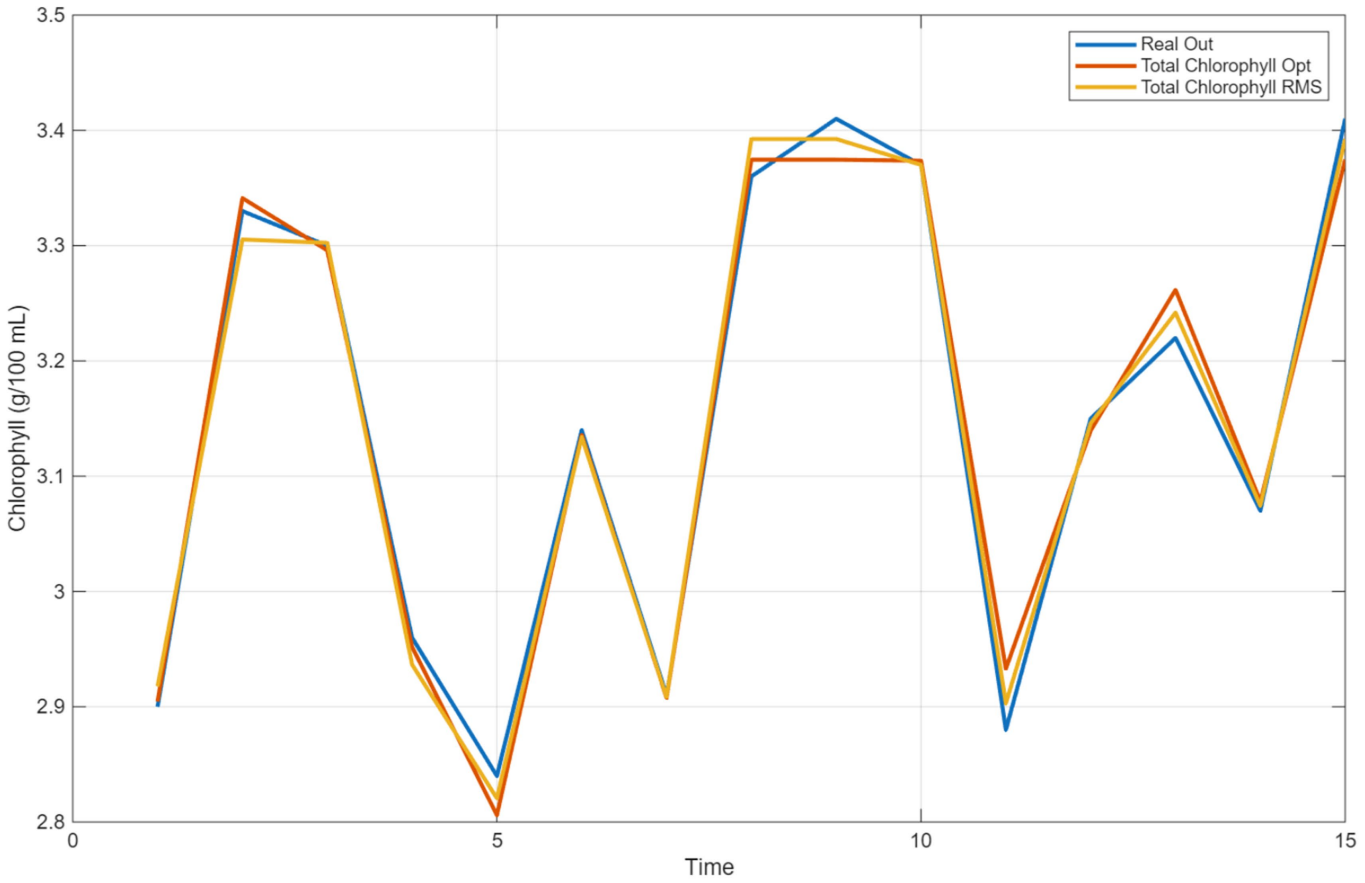
Comparison of estimation for total chrolophyll with RSM and equilibrium optimization.

Figure 3 presents the comparative performance of the EO algorithm and the RSM algorithm for the estimation of total chlorophyll concentration. The EO algorithm, represented in orange in the graph, showed an accuracy very close to the “Real Out” curve. This demonstrates that the algorithm can accurately model the system’s real behavior. Although the outputs of the RSM algorithm, shown in yellow, were generally close to the “Real Out” curve, larger deviations were observed in some regions compared to the EO optimization algorithm. In particular, the prediction performance of the RMS algorithm decreased in regions with low chlorophyll values. The *x*-axis in the graph represents the time or the order of the experimental samples, while the *y*-axis shows the amount of chlorophyll. Both algorithms follow the “Real Out” curve at specific intervals, but there are critical points where they differ from each other. In the 5th and 10th samples, the EO optimization algorithm produces results closest to the real values, while slight deviations are observed in the RSM algorithm. Both algorithms are successful in adapting to sudden changes. However, the RSM algorithm showed higher deviations compared to the EO algorithm at some sudden peaks (for example, in the 10th example). While the Equilibrium Optimization Algorithm provides more accurate and balanced results, the RSM Algorithm offers a faster and simpler calculation structure; however, it lags behind the EO algorithm in terms of accuracy. Especially at low chlorophyll levels, the algorithm’s prediction accuracy decreases.

As a result of the comparison, it can be said that the EO optimization algorithm produces more consistent results compared to the RSM algorithm. Although both algorithms are successful in capturing general trends, the EO algorithm offers a higher level of accuracy in estimating the actual values. Therefore, the EO algorithm can be considered as a more suitable option for calculating the total chlorophyll amount.

**Figure 4:**
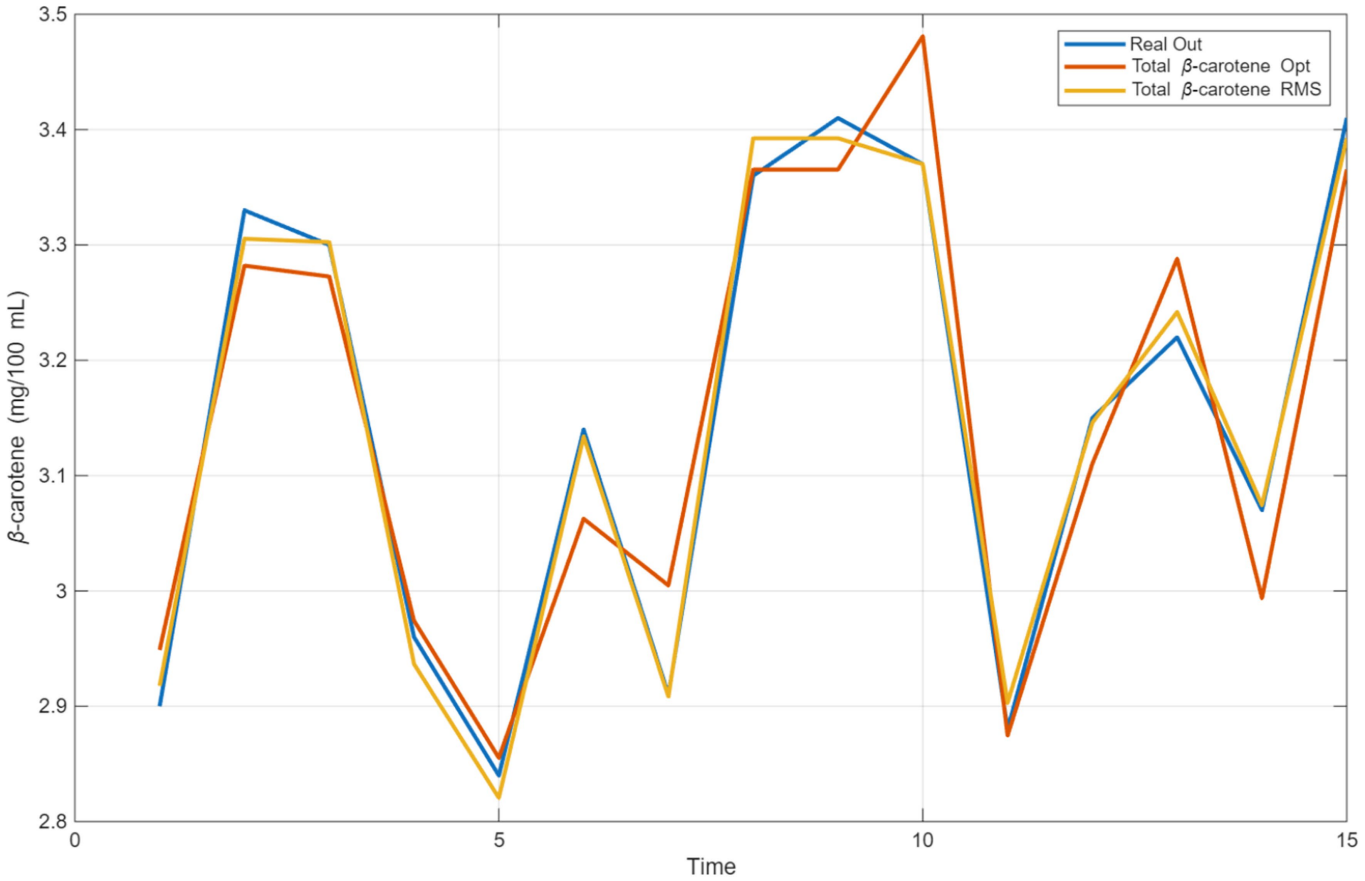
Comparison of estimation for β-karoten with RSM and equilibrium optimization.

In Figure 4, two different methods used to estimate β-carotene concentration, the equilibrium optimization algorithm and the RSM algorithm, are compared with real data. EO Algorithm (β-carotene Opt): The balance optimization algorithm, represented in orange, shows a performance very close to the “Real Out” curve. The algorithm’s prediction accuracy is particularly noteworthy at peak and trough points. The algorithm adapts to the system’s dynamic behavior and follows a trend parallel to the actual values. The RSM algorithm, highlighted in yellow, generally follows a trend similar to the “Real Out” curve. However, in some regions, particularly at points of abrupt change, larger deviations are observed compared to the equilibrium optimization algorithm. The *x*-axis in the graph represents the order of experimental samples or temporal progression, while the *y*-axis shows the β-carotene concentration. Both algorithms are successful in following general trends. However, there are differences in certain regions: For example, in the 4th and 10th examples, the balance optimization algorithm produced results very close to the actual values, while the RSM algorithm showed relatively larger deviations. At peaks (e.g., between samples 8 and 10) and troughs (e.g., sample 5) where sudden changes occur, the EO algorithm exhibited a more stable performance compared to the RSM algorithm.

As a result of the comparison, it can be concluded that the EO algorithm is a more suitable option for estimating β-carotene. While the RSM algorithm is successful in capturing general trends, the EO algorithm produces more accurate results at both peak and trough points. This shows that the EO provides a more balanced and precise prediction performance.

### 3.3 Bioactive compounds

In the study, the effect of different treatments applied to dill juice samples on the bioactive component profile was investigated, and it was determined that thermosonication application (TS-DJ) exhibited superior performance compared to other groups both at the beginning and throughout the storage process (Table 3).

**Table 3:** Changes in total chlorophyll, β-carotene, total phenolic content (TPC) and ferric reducing antioxidant power (FRAP) of dill juice samples subjected to different treatments (CDJ, P-DJ, TS-DJ) during storage.

Samples	Storageperiod (days)	Total chlorophyll (g/100 mL)	β-carotene (mg/100 mL)	TPC (mg GAE/100 mL)	FRAP (mmol TE/L)
CDJ	0	3.21 ± 0.05^aB^	35.66 ± 0.81^aB^	115.20 ± 2.88^aB^	8.92 ± 0.14^aB^
	7	3.21 ± 0.04^aB^	35.43 ± 1.11^aB^	113.33 ± 2.02^aB^	8.87 ± 0.11^aB^
	14	3.17 ± 0.05^aB^	35.27 ± 1.17^aB^	110.60 ± 3.67^aB^	8.82 ± 0.13^aB^
	21	3.16 ± 0.01^aB^	34.94 ± 0.53^aB^	110.19 ± 0.61^aB^	8.82 ± 0.03^aB^
P-DJ	0	2.96 ± 0.06^aA^	32.71 ± 0.63^aA^	102.05 ± 2.19^aA^	7.69 ± 0.15^aA^
	7	2.96 ± 0.06^aA^	32.29 ± 1.18^aA^	101.08 ± 3.68^aA^	7.70 ± 0.14^aA^
	14	2.97 ± 0.03^aA^	30.69 ± 0.94^aA^	98.21 ± 3.00^aA^	7.72 ± 0.07^aA^
	21	2.95 ± 0.03^aA^	30.77 ± 1.05^aA^	98.48 ± 3.36^aA^	7.67 ± 0.07^aA^
TS-DJ	0	3.41 ± 0.06^aC^	38.19 ± 1.18^aC^	120.30 ± 3.71^aB^	9.56 ± 0.16^aC^
	7	3.40 ± 0.06^aC^	37.73 ± 0.49^aB^	118.86 ± 1.55^aB^	9.53 ± 0.16^aC^
	14	3.38 ± 0.05^aC^	37.43 ± 0.47^aB^	117.96 ± 1.40^aB^	9.46 ± 0.14^aC^
	21	3.35 ± 0.05^aC^	37.01 ± 0.51^aC^	116.67 ± 1.66^aC^	9.38 ± 0.14^aC^

TS-DJ, thermosonication-treated dill juice; P-DJ, thermal pasteurized dill juice; CDJ, control dill juice; TPC, total phenolic content; mg GAE, milligram gallic acid equivalent; FRAP, ferric reducing antioxidant power. Results are presented as mean ± standard deviation. Different lowercase letters (a,b) within the column values indicate statistical significance between the values (*p* < 0.05). Different capital letters (A–C) within the row values indicate statistical significance between the values (*p* < 0.05).

**Figure 5:**
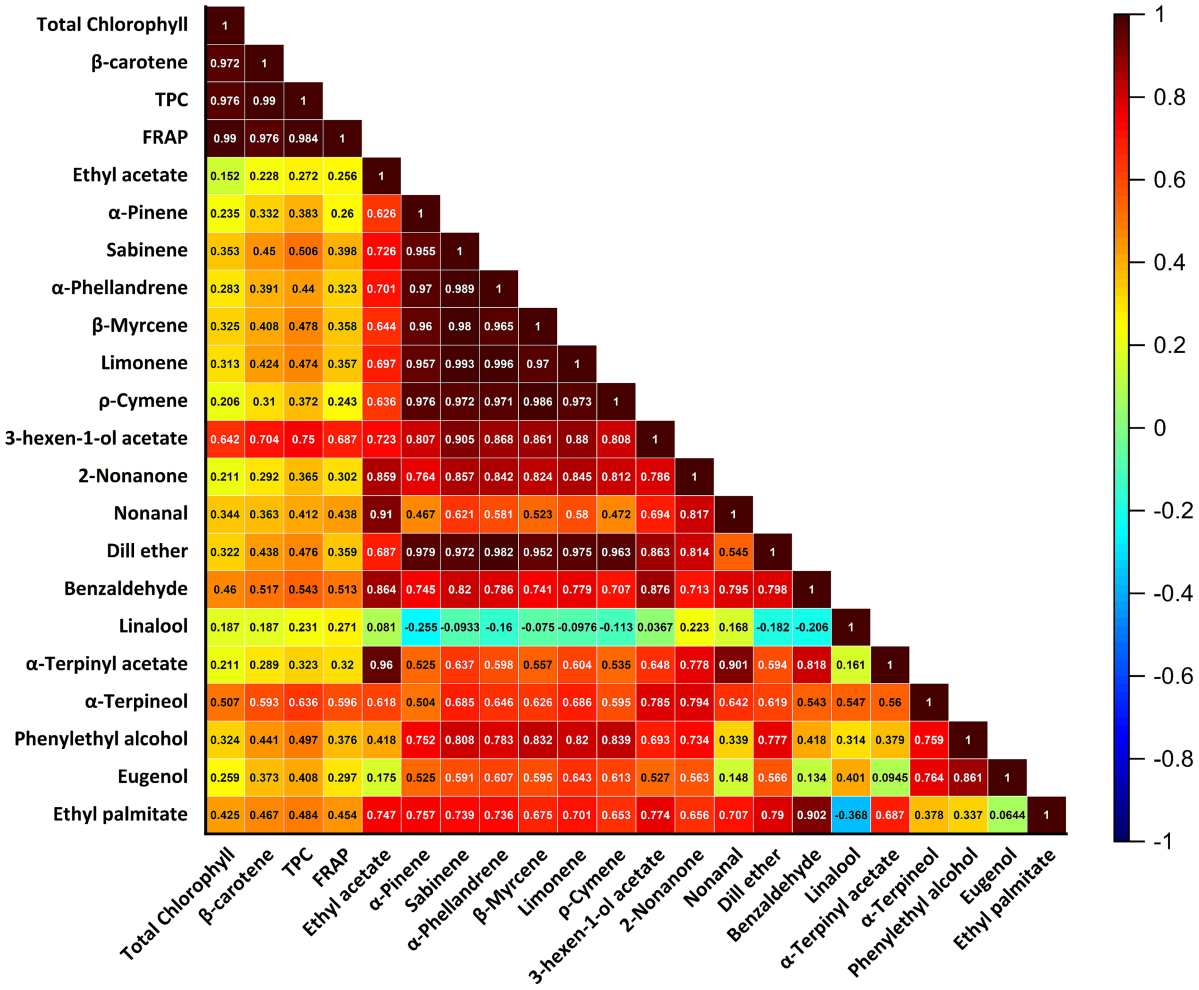
Pearson correlation matrix showing relationships among phenolic compounds, bioactive components, antioxidant capacity (FRAP), and volatile compounds in dill juice samples.

At baseline, the total chlorophyll content of the TS-DJ group was 3.41 ± 0.06 g/100 mL, β-carotene content was 38.19 ± 1.18 mg/100 mL, total phenolic substance amount was 120.30 ± 3.71 mg GAE/100 mL, and FRAP value was 9.56 ± 0.16 mmol TE/L (Table 3). These values are statistically significantly higher (*p* < 0.05) compared to both control (CDJ) and thermal pasteurized (P-DJ) groups. A study conducted on spinach juice using the thermosonication (TS) method reported that the amount of critical bioactive components, such as TPC, TFC, total flavonols, chlorophyll, and anthocyanin, was significantly increased compared to pasteurized samples. This increase supports the results of our study, showing that the TS process is more effective in enhancing the bioactive component and overall quality of the food compared to pasteurization (32). Similar results were obtained in another study conducted on freshly squeezed tomato juice. It was emphasized that the TS process has the potential to significantly increase the content of bioactive compounds such as lycopene, ascorbic acid, total phenols and flavonoids in tomato juice and improve antioxidant capacity compared to thermal pasteurization (TP) (33). Pearson correlation analysis revealed a strong and positive correlation between iron-reducing antioxidant activity and total phenolic content (FRAP) (*r* = 0.996). This demonstrates the significant contribution of phenolic compounds to antioxidant capacity. Similarly, significant positive correlations were found between total chlorophyll (*r* = 0.976) and β-carotene (*r* = 0.979) values and FRAP. These findings support the important role of these bioactive compounds in determining antioxidant properties (Figure 5). This suggests that the high bioactive levels in the TS-DJ group directly contribute to the antioxidant capacity. In particular, the use of different capitalization within the lines highlights the statistical significance of the differences between the treatments.

Decreases in bioactive components were observed in all samples throughout the storage process, but the reductions in the TS-DJ group remained minimal (Table 3). For example, in the TS-DJ group, total chlorophyll content was measured as 3.35 ± 0.05 g/100 mL, β-carotene content as 37.01 ± 0.51 mg/100 mL, TPC as 116.67 ± 1.66 mg GAE/100 mL, and FRAP value as 9.38 ± 0.14 mmol TE/L at the end of the 21st day. It was determined that these values did not differ statistically from the initial data (*p* > 0.05). According to Pearson analysis, strong positive correlations were also detected between β-carotene and aroma components such as *α*-phellandrene (*r* = 0.956), sabinene (*r* = 0.965), and α-pinene (*r* = 0.948) (Figure 5). These relationships reveal that not only the bioactive levels but also the volatile components are preserved in the TS-DJ group and progress in balance together. In contrast, more significant decreases in bioactive components occurred over time in the P-DJ and CDJ groups, indicating that thermal treatment-induced degradation hurt bioactive stability. These observations align with findings by Yıkmış et al. (34), who demonstrated that thermosonication effectively preserves carotenoids, anthocyanins, and antioxidant capacity in black carrot juice, sometimes even enhancing their levels. Similarly, studies on peach juice report superior enzyme inactivation and bioactive compound retention with thermosonication compared to thermal methods, although they caution that high amplitude, temperature, or processing time can negatively affect phenolics and ascorbic acid. This underscores the importance of carefully optimizing thermosonication parameters to maximize benefits while minimizing degradation (35).

In the overall evaluation, the thermosonication process not only provided high initial levels of bioactive components after processing but also showed superiority in maintaining the stability of these components during storage (Table 3). When evaluated based on TPC and FRAP values, it was observed that TS-DJ samples maintained their antioxidant capacity at a high level, thereby preserving their functional qualities throughout their shelf life. Pearson analysis also revealed powerful positive relationships between total chlorophyll and FRAP (*r* = 0.990) and β-carotene (*r* = 0.972) (Figure 5). These findings demonstrate that thermosonication is a more sustainable and effective alternative to traditional pasteurization techniques, supporting its applicability in the development of functional beverages. At the same time, these positive correlations between volatile components and bioactive substances suggest that both the functional and aromatic qualities of the product can be enhanced simultaneously. Non-thermal processing technologies show the potential to better preserve and improve certain quality parameters, such as bioactive profile (β-carotene, total chlorophyll, TPC, FRAP and aroma compounds) and post-digestion bioaccessibility compared to traditional thermal treatments. While studies in the literature support this potential and emphasize that care should be taken in providing appropriate process parameters, at the same time, non-thermal processing methods such as ultrasound and thermosonication give the desired quality criteria in food products and enable the development of health-rich products and have an essential place among sustainable food processing approaches (36).

### 3.4 Aroma compounds

A total of 18 aroma compounds, including 2 aldehydes, 1 ketone, 6 terpene compounds, 4 esters, and 4 alcohols, were detected in dill juice samples. The most abundant aroma compounds in dill juice samples were terpene compounds. Similar to our results (37–39), the predominant aroma compounds reported in dill samples were terpene compounds, specifically p-cymene, limonene, and α-phellandrene. In the dill juice samples evaluated in terms of aroma components (CDJ: control, P-DJ: pasteurized, TS-DJ: thermosonicated), statistically significant differences were observed depending on the applied processes and storage time (*p* < 0.05) (Table 4). Volatile compounds, such as α-pinene, α-phellandrene, and limonene, showed a general decreasing trend over time. Principally, these losses are due to oxidative reactions. In TS-DJ samples, α-phellandrene decreased from 45.29 μg/mL at the beginning to 21.04 μg/mL on the 21st day, showing a more moderate decrease compared to the other groups. This indicates that the thermosonication process may have a more protective effect against oxidative degradation of aroma compounds (Table 4). In addition, α-pinene amounts decreased over time in all groups, but the TS-DJ group exhibited higher stability compared to the P-DJ group (Table 4).

**Table 4:** Changes in total aroma components of dill juice samples subjected to different treatments (CDJ, P-DJ, TS-DJ) during storage.

Aroma data	Stroge (days)	Samples		
		CDJ	P-DJ	TS-DJ
Ethylacetate	0	1.73 ± 0.12^abB^	1.07 ± 0.11^aA^	1.25 ± 0.16^aA^
	7	1.95 ± 0.06^bcB^	1.26 ± 0.09^aA^	1.27 ± 0.10^aA^
	14	2.00 ± 0.11^cB^	1.24 ± 0.08^aA^	1.35 ± 0.09^aA^
	21	1.56 ± 0.09^aB^	1.06 ± 0.04^aA^	1.06 ± 0.11^aA^
α-pinene	0	8.99 ± 0.17^dB^	5.78 ± 0.34^cA^	6.31 ± 0.38^cA^
	7	7.73 ± 0.24^cB^	5.12 ± 0.27^bcA^	5.84 ± 0.33^bcA^
	14	6.04 ± 0.36^bB^	4.73 ± 0.39^bA^	5.37 ± 0.34^bAB^
	21	3.83 ± 0.17^aB^	2.59 ± 0.40^aA^	3.00 ± 0.18^aA^
Sabinene	0	5.39 ± 0.24^cC^	3.31 ± 0.18^cA^	4.18 ± 0.10^cB^
	7	5.13 ± 0.20^cC^	3.04 ± 0.09^bcA^	3.81 ± 0.14^cB^
	14	4.05 ± 0.24^bB^	2.67 ± 0.27^bA^	3.11 ± 0.13^bA^
	21	2.97 ± 0.18^aB^	2.08 ± 0.23^aA^	2.46 ± 0.23^aAB^
α-phellandrene	0	58.35 ± 2.10^cC^	35.86 ± 1.55^dA^	45.29 ± 1.94^dB^
	7	53.91 ± 1.04^cC^	30.90 ± 1.15^cA^	35.62 ± 0.62^cB^
	14	43.18 ± 1.73^bB^	26.45 ± 1.58^bA^	29.82 ± 0.80^bA^
	21	26.74 ± 3.36^aB^	16.32 ± 1.33^aA^	21.04 ± 1.26^aAB^
β-myrcene	0	3.58 ± 0.29^cB^	1.80 ± 0.19^cA^	2.34 ± 0.32^bA^
	7	3.13 ± 0.14^cC^	1.69 ± 0.15^bcA^	2.17 ± 0.18^bB^
	14	2.04 ± 0.13^bB^	1.35 ± 0.12^abA^	1.78 ± 0.24^abAB^
	21	1.40 ± 0.15^aB^	1.07 ± 0.08^aA^	1.24 ± 0.09^aAB^
Limonene	0	41.48 ± 1.47^cC^	24.01 ± 1.05^dA^	31.31 ± 2.55^dB^
	7	38.16 ± 0.86^cC^	20.18 ± 1.10^cA^	24.63 ± 1.06^cB^
	14	28.64 ± 1.83^bB^	15.96 ± 1.37^bA^	18.99 ± 1.15^bA^
	21	18.97 ± 1.62^aC^	9.42 ± 0.63^aA^	14.24 ± 0.74^aB^
ρ-cymene	0	7.02 ± 0.39^dB^	4.10 ± 0.37^cA^	4.53 ± 0.45^cA^
	7	5.89 ± 0.20^cC^	3.68 ± 0.18^cA^	4.27 ± 0.27^cB^
	14	4.17 ± 0.28^bB^	2.97 ± 0.15^bA^	3.34 ± 0.19^bA^
	21	3.08 ± 0.28^aB^	2.19 ± 0.12^aA^	2.20 ± 0.34^aA^
3-hexen-1-olacetate	0	5.25 ± 0.38^aB^	2.94 ± 0.18^abA^	5.11 ± 0.35^cB^
	7	5.43 ± 0.41^aC^	3.12 ± 0.12^bA^	4.35 ± 0.15^bB^
	14	4.51 ± 1.59^aA^	2.77 ± 0.23^abA^	4.26 ± 0.13^bA^
	21	3.87 ± 0.42^aB^	2.44 ± 0.23^aA^	3.06 ± 0.24^aAB^
2-Nonanone	0	0.95 ± 0.13^bB^	0.58 ± 0.09^aA^	0.67 ± 0.07^aA^
	7	0.83 ± 0.07^abB^	0.50 ± 0.03^aA^	0.58 ± 0.06^aA^
	14	0.85 ± 0.07^abB^	0.54 ± 0.06^aA^	0.59 ± 0.07^aA^
	21	0.65 ± 0.07^aA^	0.56 ± 0.08^aA^	0.53 ± 0.07^aA^
Nonanal	0	0.84 ± 0.14^abB^	0.42 ± 0.08^aA^	0.66 ± 0.11^aAB^
	7	1.04 ± 0.10^abC^	0.38 ± 0.03^aA^	0.64 ± 0.11^aB^
	14	1.15 ± 0.16^bB^	0.63 ± 0.07^bA^	0.66 ± 0.10^aA^
	21	0.76 ± 0.09^aA^	0.53 ± 0.09^abA^	0.63 ± 0.13^aA^
Dillether	0	18.12 ± 0.82^cC^	12.16 ± 0.79^cA^	13.94 ± 1.24^cA^
	7	15.59 ± 0.64^bB^	11.24 ± 0.73^cA^	12.80 ± 0.84^bcA^
	14	14.34 ± 0.63^bC^	9.26 ± 0.70^bA^	11.54 ± 0.54^bB^
	21	9.62 ± 0.92^aB^	6.47 ± 0.63^aA^	7.96 ± 0.70^aAB^
Benzaldehyde	0	4.08 ± 0.36^abB^	2.45 ± 0.46^abA^	3.74 ± 0.52^bB^
	7	5.13 ± 0.89^bB^	3.28 ± 0.33^cA^	4.13 ± 0.19^bAB^
	14	4.80 ± 0.47^abB^	2.96 ± 0.11^bcA^	3.71 ± 0.28^bA^
	21	3.30 ± 0.58^aB^	1.96 ± 0.11^aA^	2.53 ± 0.38^aAB^
Linalool	0	3.38 ± 0.40^bB^	1.73 ± 0.29^aA^	2.14 ± 0.14^aA^
	7	1.94 ± 0.57^aA^	1.54 ± 0.22^aA^	1.84 ± 0.18^aA^
	14	2.09 ± 0.28^aAB^	1.65 ± 0.12^aA^	2.23 ± 0.24^aB^
	21	4.20 ± 0.29^bB^	2.88 ± 0.13^bA^	3.40 ± 0.55^bB^
α-terpinylacetate	0	5.82 ± 0.19^aB^	3.23 ± 0.31^abA^	3.10 ± 0.34^aA^
	7	7.15 ± 0.27^bB^	3.81 ± 0.22^bA^	4.12 ± 0.32^abA^
	14	7.26 ± 0.54^bB^	3.67 ± 0.23^bA^	4.61 ± 0.58^bA^
	21	5.52 ± 0.58^aC^	2.65 ± 0.31^aA^	4.06 ± 0.19^abB^
α-terpineol	0	5.60 ± 0.74^aB^	2.93 ± 0.40^bA^	5.11 ± 0.31^bB^
	7	4.38 ± 0.44^aB^	2.48 ± 0.24^abA^	3.58 ± 0.25^aB^
	14	4.64 ± 0.48^aC^	2.05 ± 0.16^aA^	3.19 ± 0.18^aB^
	21	5.08 ± 0.36^aB^	2.99 ± 0.36^bA^	3.55 ± 0.21^aA^
Phenylethylalcohol	0	1.31 ± 0.36^bA^	0.80 ± 0.13^bA^	0.76 ± 0.21^aA^
	7	0.83 ± 0.15^abB^	0.37 ± 0.08^aA^	0.73 ± 0.11^aB^
	14	0.54 ± 0.07^aB^	0.25 ± 0.06^aA^	0.48 ± 0.08^aB^
	21	0.71 ± 0.07^aB^	0.38 ± 0.12^aA^	0.51 ± 0.07^aB^
Eugenol	0	0.93 ± 0.28^bA^	0.67 ± 0.12^bA^	0.84 ± 0.18^bA^
	7	0.49 ± 0.12^abA^	0.26 ± 0.08^aA^	0.34 ± 0.07^aA^
	14	0.36 ± 0.07^aA^	0.18 ± 0.08^aA^	0.29 ± 0.07^aA^
	21	0.60 ± 0.13^abB^	0.33 ± 0.07^aA^	0.44 ± 0.05^aAB^
Ethylpalmitate	0	1.22 ± 0.31^abA^	0.74 ± 0.12^bA^	1.03 ± 0.09^bA^
	7	1.32 ± 0.22^abB^	0.77 ± 0.10^bA^	1.17 ± 0.05^bcB^
	14	1.55 ± 0.50^bA^	0.89 ± 0.07^bA^	1.30 ± 0.08^cA^
	21	0.63 ± 0.20^aA^	0.41 ± 0.06^aA^	0.51 ± 0.09^aA^

P-DJ, thermal pasteurized dill juice; TS-DJ, thermosonication-treated dill juice; CDJ, Control dill juice; Different lowercase letters (a–d) within the column values indicate statistical significance between the values (*p* < 0.05). Results are presented as mean ± standard deviation. Different capital letters (A–C) within the row values indicate statistical significance between the values (*p* < 0.05).

When sabinene, β-myrcene, and *ρ*-cymene components were examined, it was observed that the TS-DJ group provided higher protection compared to other groups (Table 4). Notably, the sabinene content remained at a 2.46 μg/mL level in TS-DJ samples at the end of the 21st day, whereas it decreased to 2.08 μg/mL in the P-DJ group. The difference between these values is statistically significant (*p* < 0.05). Similarly, β-myrcene compound decreased to 1.40 μg/mL in the control group, while this value was maintained as 1.24 μg/mL in the TS-DJ group (Table 4). These results reveal that the TS-DJ application provides an advantage in preserving volatile compounds under fixed and controlled conditions. The beneficial effect on aroma components may be attributable to the synergistic impacts of cavitation and temperature during thermosonication treatment (40).

Dillether, benzaldehyde, and linalool components exhibited severe losses, particularly with increasing storage periods (Table 4). While the dillether amount was initially 18.12 μg/mL in the control sample, it decreased to 9.62 μg/mL at the end of the 21st day. However, this decrease was less pronounced in the TS-DJ samples (from 13.94 to 7.96 μg/mL), suggesting that thermosonication delayed the degradation of the volatile oil components. A similar situation was observed for benzaldehyde; while the TS-DJ sample was maintained at a relatively high level, 2.53 μg/mL, at the end of the 21st day, it decreased to 1.96 μg/mL in the P-DJ group. These differences were significant both in terms of time and treatment (Table 4).

Finally, when secondary aroma compounds such as *α*-terpineol, α-terpinyl acetate, and phenylethyl alcohol were examined, it was observed that the decreases in TS-DJ samples with time were lower than the other groups (Table 4). For example, α-terpineol, which was 5.11 μg/mL at the beginning, decreased to 3.55 μg/mL in TS-DJ at the end of the 21st day, whereas it was 2.99 μg/mL in the P-DJ group. This shows that the TS-DJ process contributes to aroma stability. When all components are considered, statistically significant differences (indicated by letter groups a-d and A-C) reveal substantial variations between treatments and storage days (Table 4). These findings demonstrate that thermosonication preserves aroma compounds more effectively than conventional pasteurization. Similar results were reported by Cheng et al. (41) and Yıkmış et al. (42).

### 3.5 Bioaccessibility

The level of stability and bioaccessibility of bioactive compounds throughout the digestive process is a determining factor in the effectiveness of functional food products. In this study, the preservation levels of bioactive compounds were evaluated through total phenolic substance (TPC), β-carotene, and chlorophyll contents in thermosonicated (TS-DJ), thermally pasteurized (P-DJ), and control (CDJ) dill juice samples. The findings show that TS-DJ samples retain the highest levels of bioactive compounds throughout the digestion process, indicating that the applied treatment increases the digestibility of bioactive compounds. Furthermore, correlations between these compounds indicate that changes in bioactive compound content are closely related to changes in aroma profile (Figure 5). Meena et al. (43) showed in their review that ultrasound treatment disrupts cell structures through cavitation, increasing the release of bioactive components and access to digestive enzymes while also improving the bioavailability of mineral and phytochemical compounds. The use of TS together with natural antimicrobial agents provides synergistic benefits in terms of food safety and shelf life, and future research should be conducted to optimize process parameters specific to different food matrices and make them economical and applicable (44).

When Table 5 is examined, it is observed that the TPC values are at their highest level in the TS-DJ sample before digestion, at 120.30 ± 3.71 mg GAE/L. This value is 4.4% higher than CDJ and 17.9% higher than P-DJ (*p* < 0.05). This superiority was maintained in all digestive phases. For example, at the end of the intestinal phase, while the TS-DJ sample reached a level of 34.22 ± 1.31 mg GAE/L, the P-DJ decreased to only 22.49 ± 3.07 mg GAE/L. As seen in Figure 5, robust positive correlations were observed between TPC and FRAP (*r* = 0.996), β-carotene (*r* = 0.979), and total chlorophyll (*r* = 0.976). This shows that polyphenolic structures contribute to both antioxidant capacity and other pigment components. It has been reported that the total phenolic substance content and antioxidant activity in the “Nectar” product obtained by adding pure water to the “Soursop” fruit grown in tropical regions increased significantly with the TS process, and also that *in vitro* intestinal bioaccessibility was higher compared to the control and pasteurized groups (45).

**Table 5:** Total phenolic substance (TPC) in CDJ, P-DJ and TS-PJ samples during the simulated digestion process.

Digestion phases	Storage period	TPC (mg GAE/L)		
		CDJ	P-DJ	TS-PJ
Undigested	0 day	115.20 ± 2.88^b^	102.05 ± 2.19^a^	120.30 ± 3.71^c^
	7 days	113.33 ± 2.02^b^	101.08 ± 3.68^a^	118.86 ± 1.55^b^
	14 days	110.60 ± 3.67^b^	98.21 ± 3.00^a^	117.96 ± 1.40^b^
	21 days	110.19 ± 0.61^b^	98.48 ± 3.36^a^	116.67 ± 1.66^c^
Oral digestion	0 day	90.26 ± 4.09^b^	75.49 ± 2.03^a^	97.03 ± 2.36^b^
	7 days	88.81 ± 3.90^b^	74.86 ± 5.34^a^	95.88 ± 0.93^b^
	14 days	86.64 ± 3.92^b^	72.64 ± 2.18^a^	95.14 ± 0.73^c^
	21 days	84.85 ± 2.27^b^	71.54 ± 3.16^a^	92.16 ± 1.95^c^
Gastric digestion	0 day	55.06 ± 2.49^b^	45.07 ± 2.81^a^	62.10 ± 1.51^c^
	7 days	53.87 ± 2.34^b^	44.68 ± 3.92^a^	60.40 ± 1.28^b^
	14 days	52.85 ± 2.39^b^	43.38 ± 2.92^a^	60.90 ± 0.47^c^
	21 days	50.30 ± 1.17^b^	41.32 ± 4.02^a^	58.99 ± 1.25^c^
Intestinal digestion	0 day	36.53 ± 1.91^b^	27.94 ± 1.74^a^	42.03 ± 1.69^c^
	7 days	33.18 ± 0.17^b^	26.04 ± 1.84^a^	37.23 ± 1.42^c^
	14 days	35.07 ± 1.98^b^	26.89 ± 1.81^a^	41.21 ± 0.85^c^
	21 days	30.36 ± 3.23^b^	22.49 ± 3.07^a^	34.22 ± 1.31^b^
Recovery %	0 day	31.69 ± 0.88^b^	27.38 ± 1.67^a^	34.93 ± 0.34^c^
	7 days	29.28 ± 0.45^b^	25.75 ± 1.42^a^	31.34 ± 1.61^b^
	14 days	31.70 ± 0.87^b^	27.37 ± 1.66^a^	34.90 ± 0.35^c^
	21 days	27.57 ± 3.08^a^	22.85 ± 3.11^a^	29.33 ± 1.12^a^

TS-DJ, thermosonication-treated dill juice; CDJ, control dill juice; P-DJ, thermal pasteurized dill juice; TPC, total phenolic content; mg GAE, miligram gallic acid equivalent. Values with the different letters within the line are significantly different (*p* < 0.05).

Considering the β-carotene levels (Table 6), the TS-DJ samples showed the highest values at all stages, both before and after digestion. In undigested samples, the TS-DJ sample was found to be 16.7% higher than the P-DJ sample, with a value of 38.19 ± 1.18 mg/100 mL. This difference was also preserved at the end of the intestinal phase; while the TS-DJ sample was at the level of 7.02 ± 0.52 mg/100 mL, it decreased to 4.78 ± 0.23 mg/100 mL in P-DJ. According to the Pearson analysis results, high correlations were detected between β-carotene and aroma components such as α-phellandrene (*r* = 0.956), sabinene (*r* = 0.965), and α-pinene (*r* = 0.948) (Figure 5). These findings indicate that the volatile compound profile and lipophilic antioxidants can be protected together.

**Table 6:** β-carotene in CDJ, P-DJ and TS-PJ samples during the simulated digestion process.

Digestion phases	Storage period	β-carotene (mg/100 mL)		
		CDJ	P-DJ	TS-PJ
Undigested	0 day	35.66 ± 0.81^b^	32.71 ± 0.63^a^	38.19 ± 1.18^b^
	7 days	35.43 ± 1.11^b^	32.29 ± 1.18^a^	37.73 ± 0.49^b^
	14 days	35.27 ± 1.17^b^	30.69 ± 0.94^a^	37.43 ± 0.47^b^
	21 days	34.94 ± 0.53^b^	30.77 ± 1.05^a^	37.01 ± 0.51^c^
Oral digestion	0 day	27.10 ± 0.61^b^	22.78 ± 0.07^a^	29.02 ± 0.89^c^
	7 days	26.70 ± 1.20^b^	22.29 ± 1.33^a^	28.30 ± 0.58^b^
	14 days	26.80 ± 0.89^b^	21.37 ± 0.29^a^	28.45 ± 0.36^c^
	21 days	25.98 ± 0.89^b^	21.14 ± 1.05^a^	28.00 ± 0.51^b^
Gastric digestion	0 day	15.18 ± 0.35^b^	12.38 ± 0.31^a^	16.65 ± 0.90^b^
	7 days	14.86 ± 0.67^b^	12.10 ± 0.42^a^	16.23 ± 0.76^c^
	14 days	15.01 ± 0.50^b^	11.62 ± 0.42^a^	16.31 ± 0.63^b^
	21 days	14.54 ± 0.50^b^	11.48 ± 0.49^a^	16.06 ± 0.70^c^
Intestinal digestion	0 day	6.37 ± 0.15^b^	5.11 ± 0.04^a^	7.38 ± 0.59^c^
	7 days	6.19 ± 0.21^b^	5.04 ± 0.34^a^	7.14 ± 0.51^b^
	14 days	6.30 ± 0.21^b^	4.80 ± 0.04^a^	7.24 ± 0.56^c^
	21 days	6.11 ± 0.21^b^	4.78 ± 0.23^a^	7.02 ± 0.52^b^
Recovery %	0 day	17.88 ± 0.00^b^	15.64 ± 0.35^a^	19.33 ± 1.26^b^
	7 days	17.47 ± 0.08^b^	15.61 ± 0.50^a^	18.92 ± 1.12^b^
	14 days	17.90 ± 0.00^b^	15.63 ± 0.38^a^	19.33 ± 1.25^b^
	21 days	17.48 ± 0.36^b^	15.54 ± 0.37^a^	18.96 ± 1.16^b^

TS-DJ, thermosonication-treated dill juice; CDJ, control dill juice; P-DJ, thermal pasteurized dill juice; TPC, total phenolic content; mg GAE, miligram gallic acid equivalent. Values with the different letters within the line are significantly different (*p* < 0.05).

In terms of chlorophyll content, TS-DJ samples maintained their superiority in all phases (Table 7). The total chlorophyll amount, determined as 3.41 ± 0.06 g/100 mL before digestion, remained significantly higher than the P-DJ sample during storage and digestion (*p* < 0.05). For example, as of day 21 in the gastric phase, the TS-DJ sample had a value of 1.44 ± 0.05 g/100 mL, while the P-DJ was only at the level of 1.12 ± 0.05 g/100 mL. As seen in Figure 5, there are powerful positive correlations between total chlorophyll and β-carotene (*r* = 0.972), TPC (*r* = 0.976) and FRAP (*r* = 0.99). These results support the effectiveness of thermosonication in preserving specific bioactive compounds measured in this study.

**Table 7:** Total chlorophyll in CDJ, P-DJ, and TS-PJ samples during the simulated digestion process.

Digestion phases	Storage period	Total chlorophyll (g/100 mL)		
		CDJ	P-DJ	TS-PJ
Undigested	0 day	3.21 ± 0.05^b^	2.96 ± 0.06^a^	3.41 ± 0.06^c^
	7 days	3.21 ± 0.04^b^	2.96 ± 0.06^a^	3.40 ± 0.06^c^
	14 days	3.17 ± 0.05^b^	2.97 ± 0.03^a^	3.38 ± 0.05^c^
	21 days	3.16 ± 0.01^b^	2.95 ± 0.03^a^	3.35 ± 0.05^c^
Oral digestion	0 day	2.55 ± 0.02^b^	2.26 ± 0.03^a^	2.75 ± 0.03^c^
	7 days	2.49 ± 0.10^b^	2.23 ± 0.05^a^	2.67 ± 0.13^b^
	14 days	2.52 ± 0.02^b^	2.27 ± 0.03^a^	2.73 ± 0.02^c^
	21 days	2.51 ± 0.04^b^	2.25 ± 0.04^a^	2.70 ± 0.03^c^
Gastric digestion	0 day	1.33 ± 0.02^b^	1.12 ± 0.05^a^	1.46 ± 0.06^c^
	7 days	1.30 ± 0.04^b^	1.11 ± 0.03^a^	1.43 ± 0.08^b^
	14 days	1.32 ± 0.02^b^	1.12 ± 0.06^a^	1.44 ± 0.06^c^
	21 days	1.30 ± 0.02^b^	1.12 ± 0.05^a^	1.44 ± 0.05^c^
Intestinal digestion	0 day	0.69 ± 0.02^b^	0.53 ± 0.04^a^	0.78 ± 0.03^c^
	7 days	0.65 ± 0.05^b^	0.52 ± 0.03^a^	0.77 ± 0.02^c^
	14 days	0.69 ± 0.02^b^	0.53 ± 0.05^a^	0.77 ± 0.03^a^
	21 days	0.67 ± 0.02^b^	0.53 ± 0.05^a^	0.77 ± 0.02^c^
Recovery %	0 day	21.59 ± 0.80^b^	17.95 ± 1.53^a^	22.94 ± 0.74^b^
	7 days	20.44 ± 1.77^ab^	17.69 ± 0.75^a^	22.67 ± 0.26^c^
	14 days	21.57 ± 0.75^b^	17.93 ± 1.53^a^	22.93 ± 0.78^b^
	21 days	21.17 ± 0.76^b^	17.95 ± 1.53^a^	22.79 ± 0.44^b^

TS-DJ, thermosonication-treated dill juice; CDJ, control dill juice; P-DJ, thermal pasteurized dill juice; TPC, total phenolic content; mg GAE, miligram gallic acid equivalent. Values with the different letters within the line are significantly different (*p* < 0.05).

When the recovery (%) values were evaluated, TS-DJ samples had the highest post-digestion usability for all three parameters (TPC, β-carotene, and chlorophyll). For example, as of day 0, recovery rates were 34.93 ± 0.34% for TPC, 19.33 ± 1.26% for β-carotene, and 22.94 ± 0.74% for chlorophyll. These rates are 27.5, 23.9 and 21.8% higher than the P-DJ samples, respectively. According to the correlation matrix, aroma components such as α-terpineol (*r* = 0.759), phenylethyl alcohol (*r* = 0.861), and eugenol (*r* = 0.744) also show strong relationships with both FRAP and chlorophyll levels (Figure 5). In this context, the high preservation of certain bioactive components measured in our study directly contributes to both the functional and aromatic quality of the product. According to a study conducted on Jamun fruit milk dessert, apart from fruit or vegetable juices, it is reported that the TS process increases the digestibility and bioaccessibility of nutrients through physical changes in the food matrix, such as changes in fat droplet size and the release of bioactive compounds (40). At the same time, in conjunction with various studies reviewed, including the study by Yıkmış et al. (46), it is supported that TS application can increase the bioaccessibility of bioactive compounds naturally found in foods.

### 3.6 Distinguishing processing effects using PCA and cluster analysis

The effects of different processing techniques applied to dill juice samples were comprehensively evaluated through multivariate statistical analyses. The Principal Component Analysis (PCA) biplot presented in part (A) shows how the sample groups are positioned on the component axis. It is observed that the samples are separated from each other in terms of the first two principal components, which collectively explain a significant portion of the total variance. The TS-DJ (Ultrasound + Microwave) sample is positioned along the positive PC1 and PC2 axes in the PCA space, and this position reveals its strong positive relationship with bioactive parameters, especially total chlorophyll, total phenolic content, β-carotene, and FRAP. This combined treatment is an important finding indicating that the functional properties of the product may be increased with the higher retention of bioactive compounds observed in the study and their preserved bioaccessibility after digestion. In contrast, the P-DJ (thermal pasteurization) sample is positioned more toward the negative axis, indicating lower values, particularly regarding pigment and antioxidant capacity. The CDJ (control) sample is located in a more central position in the PCA plane, indicating that the untreated sample exhibits balanced but limited variation in terms of parameters.

**Figure 6:**
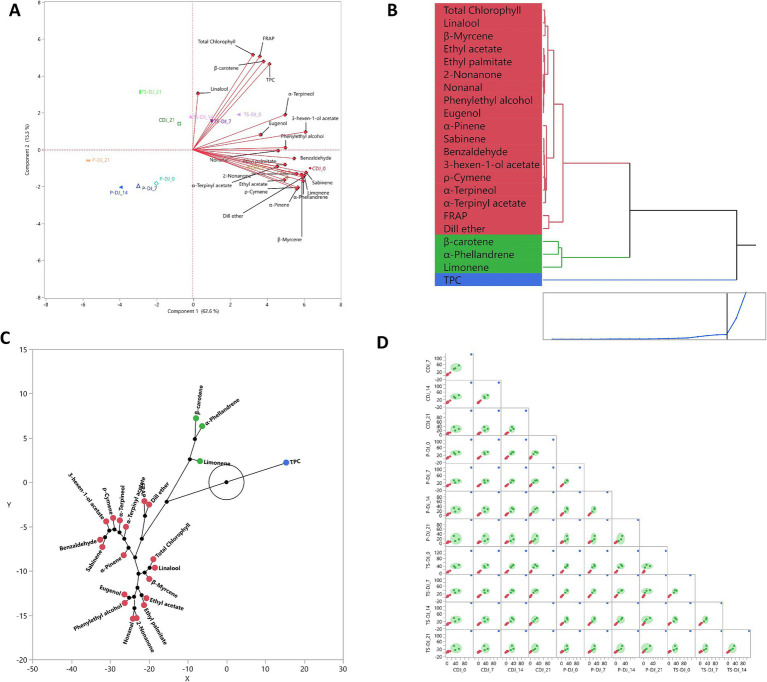
Multivariate statistical evaluations of dill juice samples processed with different techniques. **(A)** The PCA biplot illustrates the distribution and relationships between treatments and quality-related variables. **(B)** The hierarchical clustering heatmap groups bioactive and volatile compounds based on similarity patterns. **(C)** The constellation plot reveals the spatial associations and clustering tendencies of the compounds, as influenced by the treatments. **(D)** The scatterplot matrix shows pairwise correlations and distribution patterns among key chemical and functional attributes.

According to the hierarchical clustering analysis presented in part (B), the similarities between the samples and chemical components are visualized. In particular, the TS-DJ sample is located in a separate cluster, indicating that the combined process gives the sample a unique profile. As a result of this clustering, it is evident that the TS-DJ group exhibits a homogeneous structure, particularly in terms of antioxidant capacity, phenolic compounds, and specific volatile components. (C) The relationships between the samples and chemical components were evaluated on the spatial plane with the constellation plot, and it was observed that compounds exhibiting similar behavior were grouped into clusters. Specific to the TS-DJ process, the close association of certain bioactive and aromatic compounds suggests that these compounds increase together, exhibiting synergistic effects after the process. This supports the possibility of potential correlative or co-expression between functional components. (D) Scatterplot Matrix analysis provides analysis of bidirectional relationships and distribution patterns. In particular, statistically significant positive relationships were observed between total phenolic substances and FRAP, and total chlorophyll and β-carotene, indicating that these components act together and respond similarly to processing conditions. These multifaceted evaluations strongly suggest that the TS-DJ process provides significant improvement effects on the sample, not only individually but also in the context of the inter-compound relationships (Figure 6).

## 4 Conclusion

This study demonstrated that thermosonication is an effective method for preserving specific bioactive compounds in herbal beverages, such as dill juice, thereby enhancing their post-digestion bioaccessibility. Thermosonication ıt successfully maintained high levels of total β-carotene, chlorophyll, and phenolic content while significantly enhancing antioxidant capacity as measured by FRAP. Analyses using an *in vitro* digestion model demonstrated that thermosonication improved the solubility and stability of specific bioactive compounds, such as total β-carotene, phenolic content, TPC, and chlorophyll, during digestion. The optimization models developed using RSM and EO algorithms confirmed the reliability and practical applicability of the identified processing parameters. Additionally, thermosonication helped maintain the stability of volatile aroma compounds better than conventional thermal treatments. Overall, the results indicate that thermosonication is a promising approach to enhance the retention and bioaccessibility of bioactive compounds in plant-based beverages. In this respect, the study contributes to expanding the understanding of food processing techniques for enhancing product quality.

## Data Availability

The raw data supporting the conclusions of this article will be made available by the authors, without undue reservation.
